# A new portable Fresnel magnifying loupe for nystagmus observation: a clinical education and clinical practice setting study

**DOI:** 10.1186/s12909-023-04466-z

**Published:** 2023-06-24

**Authors:** Reiko Tsunoda, Hiroaki Fushiki, Ryozo Tanaka, Mayumi Endo

**Affiliations:** 1 Department of Speech, Language and Hearing Therapy, Faculty of Health Sciences, Mejiro University, 320 Ukiya, Iwatsuki-Ku, Saitama-Shi, Saitama, 339-8501 Japan; 2 Department of Physical Therapy, Faculty of Health Sciences, Mejiro University, Saitama, Japan; 3 Division of Otolaryngology, Mejiro University Ear Institute Clinic, Saitama , Japan

**Keywords:** Fresnel magnifying loupe, Frenzel goggles, Nystagmus, Student practice, HINTS

## Abstract

**Background:**

Dizziness is a common complaint of patients treated by primary care physicians. It is predominantly caused by peripheral vestibular disorders; however, central nervous system disorders should be excluded. Examination of the eye movements and nystagmus can help differentiate the disorders of the central nervous system from the peripheral vestibular disorders; however, it is often not performed appropriately. In medical education practice, nystagmus observation may facilitate an understanding of vestibular function and nystagmus characteristics. Thus, we proposed a medical education practice to master nystagmus observation using a recently developed portable Fresnel magnifying loupe that could be shielded by one eye.

**Methods:**

Thirty-three students from the Department of Physical Therapy and the Department of Speech, Language, and Hearing Therapy of the Mejiro University participated in this study. Postrotatory nystagmus was measured and compared using the new loupe and control methods, namely the naked eye and Frenzel goggles; we rated the ease of visibility using a five-point scale.

**Results:**

The number of detected cases of nystagmus was significantly higher with the new loupe than with the naked eye (*p* = 0.001). In addition, there were no significant differences in the nystagmus counts between the observations using the new loupe and Frenzel goggles (*p* = 0.087). No significant difference was observed in the visibility of eye movements between the loupe and naked eye (*p* = 1.00). The Frenzel goggles provided better visibility compared to that by the loupe (*p* = 0.034); however, none of the participants reported poor visibility using any of these methods.

**Conclusions:**

Our newly developed Fresnel loupe allows for the observation of nystagmus counts a level of reduction in fixation suppression similar to that of Frenzel goggles in an educational practice setting. Furthermore, it enables the detection of significantly more nystagmus counts compared to that by the naked eye. It offers several advantages over Frenzel goggles, including its lightweight, thin, durable, and portable design. Additionally, the loupe does not rely on a power source and can be used under normal room lighting conditions.

**Trial registration:**

This study was approved by the Medical Research Ethics Committee of Mejiro University (approval number: 21medicine-021).

## Background

Dizziness, which is a common complaint among patients treated by primary care physicians, affects approximately 20% to 30% of the general population [1]. Clinical education and practice in dizziness and balance disorders require students to understand the physiology of the vestibular system and evaluate eye movements, posture, and gait function. Peripheral vestibular disorders are the most common causes of dizziness; however, central nervous system disorders must be excluded. Particularly, a series of oculomotor-vestibular function assessments termed the Head Impulse Test-Nystagmus-Test of Skew (HINTS) is central to differentiating stroke from peripheral vestibular disorders in acute vertigo; each student is required to adopt this skill in the clinical setting [2]. Further, HINTS is termed as a three-step bedside oculomotor examination. The Head Impulse Test (HI) requires no tools; N (nystagmus) is observed with medical devices, such as the Frenzel goggles or infrared video nystagmus goggles; and TS (Test of Skew) requires the shielding of one eye.

We considered it necessary to observe actual nystagmus in medical education practice to understand its features. The caloric and rotary chair tests induce nystagmus in healthy individuals. These vestibular function tests are performed in clinical settings and are safe. The rotary chair test is less uncomfortable than the caloric test; particularly, the postrotational nystagmus test is a rapid and easy method to observe stable peripheral nystagmus. Peripheral nystagmus, including postrotatory nystagmus, is weakened by gazing; therefore, nystagmus is observed in clinical examinations using medical devices that suppress gazing [3]. The Frenzel goggles are used as nystagmus observation devices, which incorporate a high-diopter lens that makes it difficult for the patient to focus on the surroundings; however, the examiner can observe the patient's magnified eyes distinctly when the light shines from inside the goggles [4]. However, the Frenzel goggles are relatively expensive for individual student use, and nystagmus caused by rotational stimuli is observed with the naked eye in student practice. This attribute necessitates a simple and inexpensive tool that can observe nystagmus better than the naked eye.

## Methods

### Aim

In this study, we aimed to propose a medical education practice to observe postrotatory nystagmus using a recently developed portable Fresnel magnifying loupe to master the technique of nystagmus observation.

### Participants

Thirty-four students from the Department of Physical Therapy and the Department of Speech, Language, and Hearing Therapy of the Mejiro University participated in the study. Previously, they had received a lecture on the characteristics and direction of spontaneous nystagmus. However, they observed nystagmus for the first time. Participants were informed regarding the purpose and methods of the study in writing, and their written consent for participation was obtained.

### Designing and manufacturing of the Fresnel magnifying loupe

**Fig. 1:**
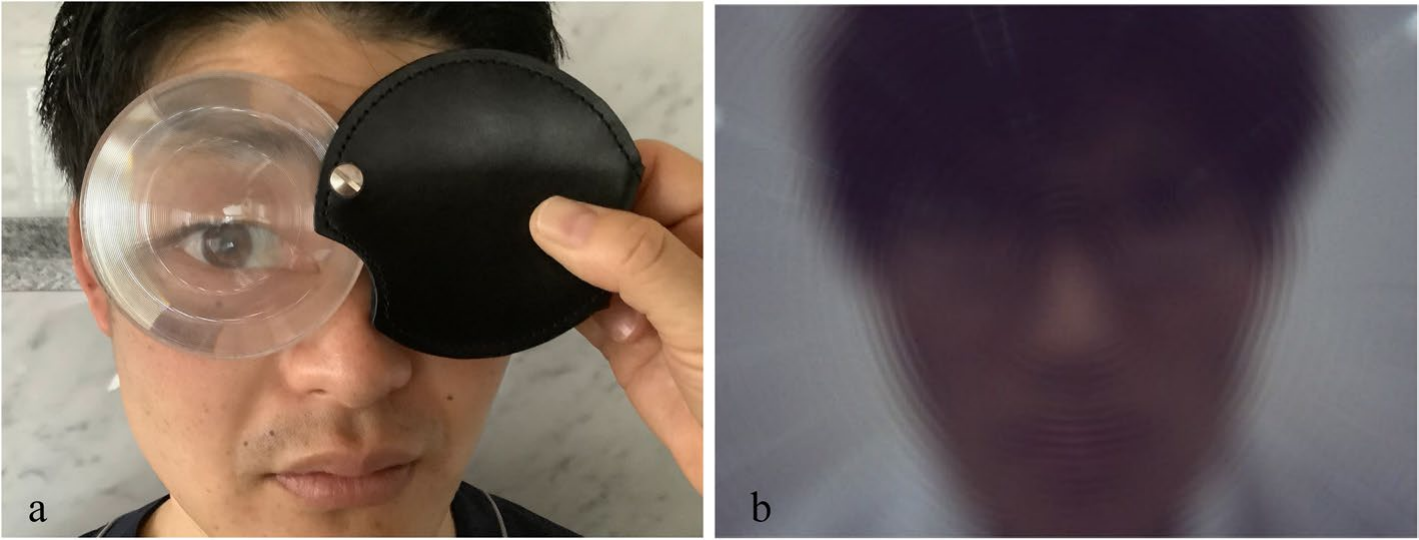
The Fresnel-based loupe. **a** The Fresnel plastic lens allows magnification of the participant’s eyes for observation. **b** Visibility from the participants. The surroundings appear blurred

**Fig. 2:**
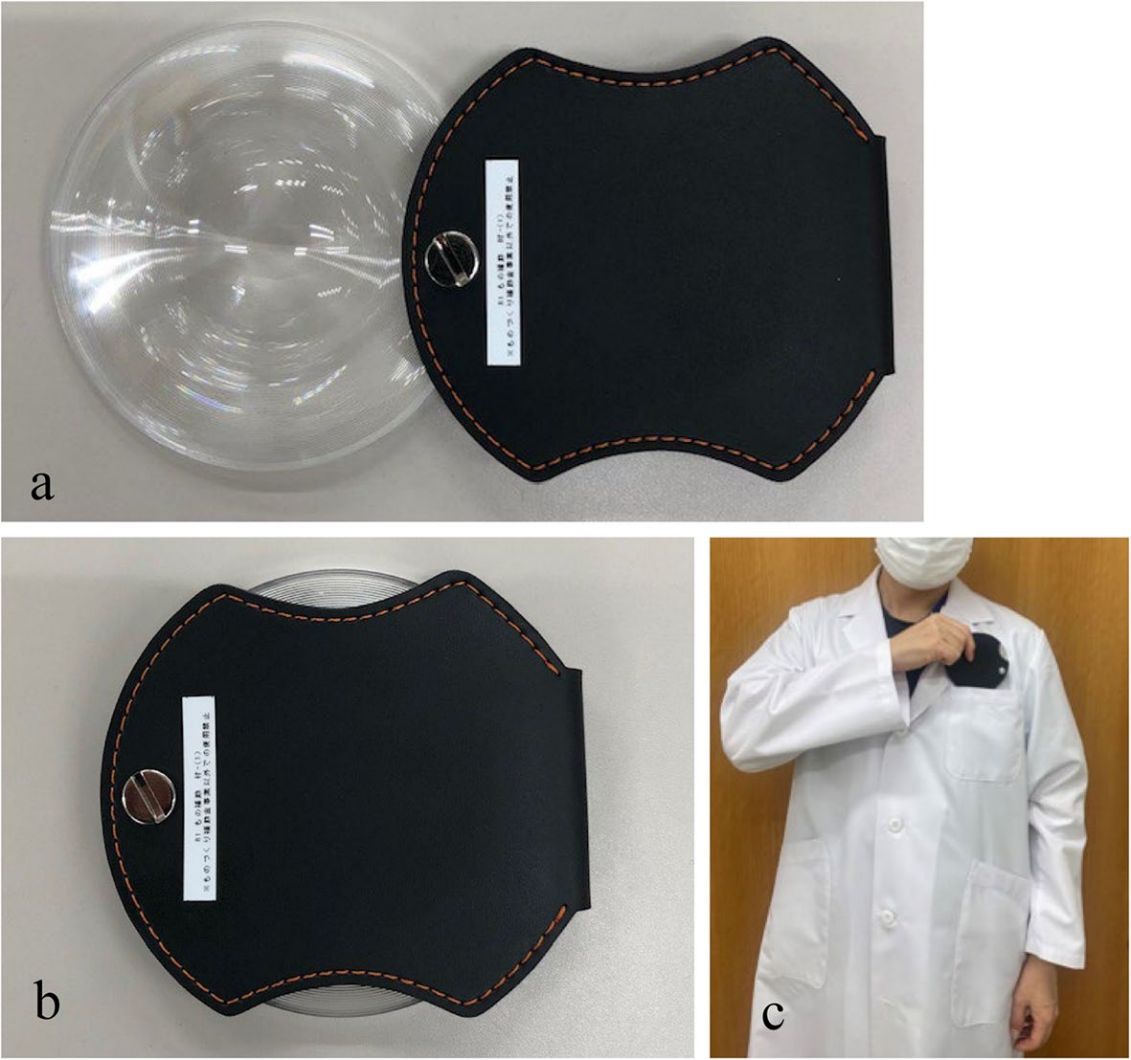
The size of the Fresnel-based loupe. **a** Dimensions used are 160 × 90 × 10 mm. **b** The stored size is 90 × 90 × 10 mm. **c** The loupe can be carried in the breast pocket

We used a new loupe for the nystagmus observations (Fig. 1a). It consists of a thin Fresnel plastic lens and storage cover (Fig. 2a, b). Upon placing the lens in front of one eye and hiding the other eye by a storage cover, the observer can visualize the participant’s eye magnified through the Fresnel lens; however, the participant cannot visualize the surroundings (Fig. 1b) and the gaze remains suppressed. It does not require a power supply and can be used under normal room light. The focal distance of the loupe was 50 mm at 2 × magnification. It weighed 24 g and was only 10-mm thick while storing the lens, thus making it portable (Fig. 2c). Owing to its simple construction, the loupe can be offered at a lower cost than the conventional Frenzel goggles. All the parts were disinfected with alcohol.

### Postrotatory nystagmus

The students were divided into groups of three and alternated as the participant, observer, and chair turner. The participants sat in an office swivel chair with eyes closed and the head bent forward at 30°. The chair-turner rotated the chair five times at a rate of 1 rotation/s and rapidly stopped the chair. They opened their eyes immediately, and the observer measured the nystagmus counts using the new loupe and a control method, namely the naked eye or Frenzel goggles. While observing the new loupe, one eye was covered by the lens, whereas the other was hidden by its storage cover to suppress gazing. Eighteen students compared the loupe with the naked eye, whereas 16 students compared it with the Frenzel goggles. Each participant performed a rotation test in both directions, and a total of four observations were performed at intervals of at least 15 min. The order of observations was random.

### Visibility of nystagmus

Further, nystagmus visibility was evaluated using a questionnaire. The observers used a five-point scale (1 = very good, 2 = good, 3 = moderate, 4 = less good, and 5 = poor) to determine whether they could visualize the eyes distinctly with this loupe and a control method. Observations were conducted in a student practice room with ceiling lighting, and the brightness was comparable to that in an outpatient office.

### Data analysis and statistics

Statistical analysis was performed using the 2-sided Wilcoxon signed-rank method, and we considered *p-*value < 0.05 as a significant difference (Bell Curve for Excel Ver.3).

## Results

### Postrotatory nystagmus

**Fig. 3:**
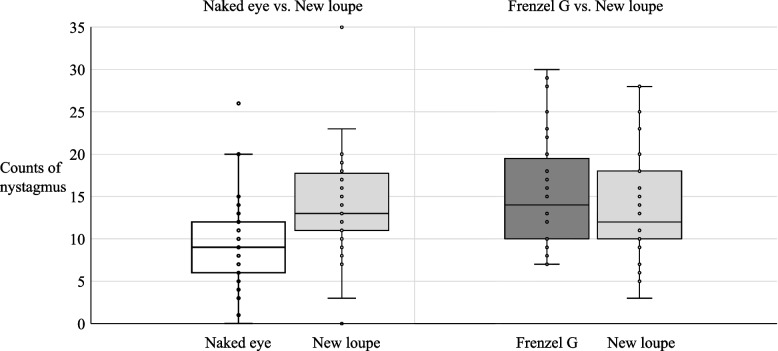
Comparison of nystagmus counts (New loupe vs. control method). Significantly higher number of cases of nystagmus are observed with the new loupe (M13, LQ11, and HQ17.8) than that with the naked eye (M 9, LQ6, and HQ12). However, no significant difference was observed in the nystagmus count between the new loupe (M12, LQ10, and HQ18) and Frenzel goggles (M 14, LQ10, and HQ19.5). Frenzel G, Frenzel goggles; M, median; LQ, lower quartile; and HQ, higher quartile

Eighteen students compared the naked eye to the new loupe and counted the postrotatory nystagmus for both rotations (*n* = 36). The median postrotatory nystagmus by the naked eye and new loupe was 9 (lower quartile 6, higher quartile 12) and 13 (lower quartile 11, higher quartile 17.8), respectively. Significantly more nystagmus was observed with the new loupe than that with the naked eye (*p* = 0.001). In contrast, 16 students compared the Frenzel goggles to the loupe and counted the postrotatory nystagmus for both rotations (*n* = 32). The median postrotatory nystagmus of the Frenzel goggles and the new loupe was 14 (lower quartile 10, higher quartile 19.5) and 12 (lower quartile 10, higher quartile 18), respectively. They observed no significant difference in the nystagmus count between the loupe and Frenzel goggles (*p* = 0.087) (Fig. 3).

### Visibility of nystagmus

**Fig. 4:**
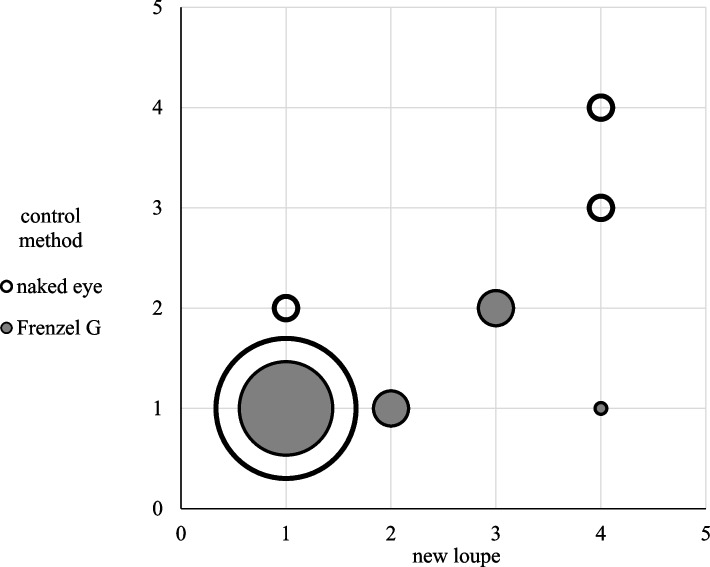
Visibility of new loupe and control method. Participants were evaluated on a 5-point grading system to assess their ability to visualize the eyes clearly with the new loupe and the control method. None of the participants reported poor (5) visibility when using either of these methods. No significant difference in visibility is observed between the loupe and naked eye (*p* = 1.00). The Frenzel goggles display better visibility than the loupe (*p* = 0.034). Grading system: 1 = very good, 2 = good, 3 = moderate, 4 = less good, and 5 = poor

No significant difference occurred in the visibility of the eye movements between the loupe and the naked eye (*p* = 1.00). The Frenzel goggles (*p* = 0.034) displayed significantly better visibility than the new loupe (Fig. 4). None of the participants reported poor visibility using any of these methods.

## Discussion

Clinical examination of eye movements and nystagmus allows the diagnosis of possible brainstem- or cerebellar lesions in several cases of dizziness and can differentiate among peripheral, central oculomotor, and vestibular lesions [5]. Dizziness is a common presentation in the emergency department, and nystagmus evaluation and a definition of its characteristics by the emergency physician are important for determining acute dizziness. One of the crucial clinical signs for differentiating between acute peripheral and central vertigo is the suppression of spontaneous nystagmus by visual fixation [6]. However, Kerber et al. examined emergency department medical records and demonstrated that despite frequent nystagmus assessment recordings in patients presenting with acute vertigo, the details generally do not allow meaningful inferences. Furthermore, the effects of the fixation removal were not mentioned. The recorded descriptions often conflict during a peripheral vestibular diagnosis [7]. The Frenzel goggles are not used in the emergency department, despite their ruggedness and portability. The underutilization of nystagmus information may be attributed to the fact that medical education programs do not incorporate up-to-date training of nystagmus assessment into their curricula. Furthermore, medical students perceive neurology as the most difficult subspecialty because of the complicated and difficult clinical examination [8]. Recently, most neuro-otology clinics and specialists have used infrared video nystagmus goggles for greater precision and permanent measurable records [3]. Therefore, students and residents may perceive the distinct nystagmus on the infrared video recordings observed in the lecture to be relatively different from that observed in the emergency department using the Frenzel goggles or the naked eye. Thus, students should be trained to observe nystagmus using the Frenzel goggles or a comparable tool.

In this study, we proposed a medical education practice of nystagmus observation for students using a new portable Fresnel magnifying loupe that is inexpensive and easy to operate. The number of nystagmus counts observed with the new loupe was significantly higher than that observed with the naked eye, despite there being no significant difference in the visibility of eye movements when using the loupe and the naked eye. These findings indicate that the new loupes prevent nystagmus attenuation caused by fixation. As previously mentioned, the assessment of visual suppression is important in distinguishing between peripheral and central nystagmus during observation. In this context, the utilization of loupes may prove beneficial in clinical settings. Because this study was conducted with physical therapy and speech-language and hearing therapy students, even residents, physicians, and medical staff unfamiliar with nystagmus findings could easily observe nystagmus with the new loupe.

**Fig. 5:**
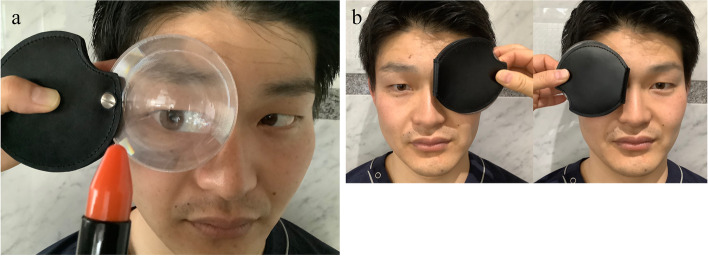
Observation of gazing nystagmus and the Test of Skew. **a** Gaze nystagmus can be observed upon instructing the participants to look at the end of a pointer without covering one eye. **b** Skew deviation is detected by the alternate cover test. The vertical position change of the unshielded eye is examined upon shielding one eye

Furthermore, students could use this loupe for HINTS training (Fig. 5). Students can perform a cover test and Test of Skew upon storing the lens in a cover (Fig. 5b). The HINTS exam has a higher sensitivity than hyperacute magnetic resonance imaging in diagnosing posterior circulation stroke in patients presenting with acute vestibular syndrome [2]. However, Mahmud et al. noted that the HINTS was sometimes inappropriately applied or incorrectly interpreted in the emergency department of a large tertiary hyperacute stroke center [9]. Therefore, adequate HINTS training should be provided in student practice, and the developed loupe will prove useful in this context.

Strupp et al. created Fresnel-based devices with 2 × (134-mm focal distance) and 4 × (63-mm focal distance) magnifications and compared them with the Frenzel goggles. The maximum slow-phase velocity of the postrotatory nystagmus with the 4 × Fresnel-based device was similar to that with the Frenzel goggles; however, the maximum slow-phase velocity was significantly lower with the 2 × device [10]. Their glasses weighed 6 g and could be fastened to the participant's nose such that both the hands of the examiner remained unoccupied. Our loupe is more hygienic because it does not contact the participant, although the examiner must secure it in place using one hand.

Yeolekar et al. reported on the usefulness of modified Google Cardboard as the Frenzel goggles, which are superior to the naked eye in identifying spontaneous nystagmus. Although it is a cheaper alternative, modified Google Cardboard should be used in a well-lit room, as it does not have inbuilt lighting [11]. Our loupe can be used with natural light and does not require a power supply.

One advantage of the new loupe is that it does not contact the face; therefore, there is less concern about clouding [10]. The Frenzel goggles cover the eyes in close contact with the participant's face. Further, the participant’s body heat causes occasional lens clouding when the room temperature is low.

The major advantages of the Fresnel magnifying loupe are as follows: First, it weighs only 24 g, is thin, and can be stored in a cover to prevent damage to the lens, thus making it portable. Second, it does not require a power supply and can be observed under normal room light. Third, the device is excellent for infection control because it can be used without contact with the participant and can be disinfected by wiping it with alcohol. Their simple structure is inexpensive, compared with the conventional Frenzel goggles. Further, it can be prepared for the practical use by numerous students.

## Limitations

This study was designed for medical education practice, which led us to utilize an office swivel chair for rotational stimulation; consequently, the rotational stimulus was not constant. Additionally, the evaluation in this study focused solely on nystagmus counts, prioritizing observability. To assess the clinical effectiveness of the new loupe in the future, it would be essential to evaluate the nystagmus frequency as well as its slow-phase velocity.

## Conclusions

The newly developed Fresnel loupe proved to be a valuable tool in practice, aiding students in their comprehension of the vestibular system. As students rotated their own chairs and observed physiological nystagmus, the loupe facilitated greater number of nystagmus counts compared to that by the naked eye observation by reducing the effects of nystagmus suppression due to visual fixation. The new loupe is easy to use, always available, light, thin, portable in a lab coat pocket, and inexpensive. Therefore, this important examination tool can be not only used in student practice but also in all environments worldwide in the future.

## Data Availability

The datasets used and/or analyzed during the current study shall be made available from the corresponding author upon reasonable request.

## Abbreviation

HINTS: Head Impulse Test-Nystagmus-Test of Skew
